# Biomechanical analysis and modeling of different vertebral growth patterns in adolescent idiopathic scoliosis and healthy subjects

**DOI:** 10.1186/1748-7161-6-11

**Published:** 2011-05-23

**Authors:** Lin Shi, Defeng Wang, Mark Driscoll, Isabelle Villemure, Winnie CW Chu, Jack CY Cheng, Carl-Eric Aubin

**Affiliations:** 1 Department of Imaging and Interventional Radiology, The Chinese University of Hong Kong, N.T., Hong Kong; 2 Mechanical Engineering Department, École Polytechnique de Montréal, Montréal, Quebec, Canada; 3 Research Center, Sainte-Justine University Hospital Center, 3175 Cote Sainte-Catherine Road, Montréal, QC H3T 1C5, Canada; 4 Department of Orthopaedics and Traumatology, The Chinese University of Hong Kong, N.T., Hong Kong

**Keywords:** finite element model, growth profile of the vertebral body, adolescent idiopathic scoliosis, bone growth modulation, scoliosis pathomechanism

## Abstract

**Background:**

The etiology of AIS remains unclear, thus various hypotheses concerning its pathomechanism have been proposed. To date, biomechanical modeling has not been used to thoroughly study the influence of the abnormal growth profile (i.e., the growth rate of the vertebral body during the growth period) on the pathomechanism of curve progression in AIS. This study investigated the hypothesis that AIS progression is associated with the abnormal growth profiles of the anterior column of the spine.

**Methods:**

A finite element model of the spinal column including growth dynamics was utilized. The initial geometric models were constructed from the bi-planar radiographs of a normal subject. Based on this model, five other geometric models were generated to emulate different coronal and sagittal curves. The detailed modeling integrated vertebral body growth plates and growth modulation spinal biomechanics. Ten years of spinal growth was simulated using AIS and normal growth profiles. Sequential measures of spinal alignments were compared.

**Results:**

(1) Given the initial lateral deformity, the AIS growth profile induced a significant Cobb angle increase, which was roughly between three to five times larger compared to measures utilizing a normal growth profile. (2) Lateral deformities were absent in the models containing no initial coronal curvature. (3) The presence of a smaller kyphosis did not produce an increase lateral deformity on its own. (4) Significant reduction of the kyphosis was found in simulation results of AIS but not when using the growth profile of normal subjects.

**Conclusion:**

Results from this analysis suggest that accelerated growth profiles may encourage supplementary scoliotic progression and, thus, may pose as a progressive risk factor.

## Background

Adolescent idiopathic scoliosis (AIS) is a 3D spinal deformity with unknown etiology [1]. Often, spinal column overgrowth during the peripubertal period is observed in AIS patients [2,3]. Correspondingly, others reported scoliotic spines to be longer than control subjects (particularly in the thoracic segments) [4], progression of scoliotic spinal deformity occurs during the adolescent growth spurt [5-7], and curve progression is correlated with the rapid spinal growth period [8]. Adolescents with the most common type of thoracic scoliosis were also found to be taller, leaner, and with hypokyphotic thoracic spines when compared to normal subjects [9,10]. In particular, the anterior spinal column was found to have relative overgrowth in AIS over normal subjects [11]. MRI studies have further confirmed the presence of longer vertebral column lengths both in AIS with thoracic or thoracolumbar curves without any corresponding changes in spinal cord length [12,13].

Many studies have reported significant differences in the pattern of growth and growth velocity between AIS and normal adolescents [9,10,14]. The mean age and the magnitude of peak sitting height growth velocity were also found to differ significantly between girls that finally progressed to scoliosis and those that did not [9]. Hägglund et al. observed above average height in scoliotic girls two years before the onset of the pubertal growth spurt [14]. In addition, radiographs of 274 AIS patients between the age of 6.5~18.5 compared to 212 age-matched controls demonstrated an early start and later cessation of the pubertal spinal growth spurt in AIS patients [10]. Stokes also documented a different growth profile in AIS patients compared to controls [15].

Based on the Hueter-Volkmann law for bone growth modulation, the "vicious cycle" qualitatively explained the mechanism of scoliotic progression in an iterative manner: the asymmetrical stress distribution leads to asymmetrical growth, which in turn causes the vertebral wedging and contributes to the spinal deformity [16]. Stokes quantitatively modeled the effect of loading asymmetry in scoliotic spines on the rate of scoliotic progression to confirm the plausibility of the "vicious cycle" principle [15]. Plaats et al. and Azegami et al. simulated the 'buckling' effect on the progression of scoliosis and showed that, on its own, buckling will not initiate scoliosis [17,18].

Finite element modeling (FEM) is an effective and objective technique that allows the direct investigation of variables of interest and can be used to test different pathomechanical hypotheses [19-22]. Villemure et al. tested the contribution of different pathogenesis hypotheses related to initial asymmetrical loads in scoliotic progression [22]. Huynh et al. demonstrated that the asymmetry of pedicle growth rate alone will contribute neither to the initiation nor the progression of the scoliotic deformity [20]. Driscoll et al. tested the influences of concave-convex biases on the progression of scoliotic curves using a FEM integrating the anterior spine and a detailed representation of growth physiology and dynamics [23], and found that concave-convex biases are potential factors that influence the progression of scoliotic curves. Until now, proper biomechanical modeling has not been used to study in depth the influence of the abnormal growth profile on the pathomechanism of curve progression in AIS.

The purpose of this study is to explore the hypothesis that the progression of AIS curve deformity, during the peripubertal period, may result from abnormal differential growth profiles of the vertebral column in AIS when compared to normal adolescent controls.

## Methods

### Finite Element Model

**Figure 1:**
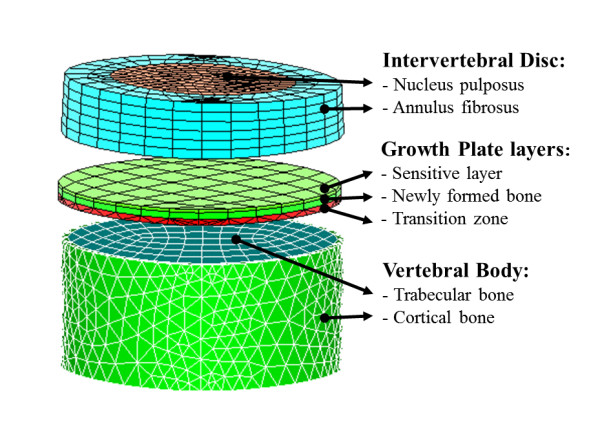
**The detailed modeling of intervertebral disc, growth plate layers, and the vertebral body in the spinal column model**.

The shape of a normal spine was used as an initial geometry and reconstructed from the bi-planar radiographs of a non-pathological female subject [24]. This geometric model is composed of 17 vertebral bodies from T1 to L5, and 16 intervertebral discs using published linear material properties (Table 1) [25]. Each vertebra was modeled as a wedged cylinder that consists of cortical and trabecular bone and three layers of the vertebral growth plates: the sensitive layer, the newly formed bone layer, and the transition layer [25,26] (Figure 1). The intervertebral disc includes the annulus fibrosus and nucleus pulposus. In this modeling approach, the sensitive layer of the vertebral growth plate receives the stress used to determine the local bone growth rate. The newly formed bone layer is where new bone is simulated (bone calcification). The transition layer connects the sensitive and the newly formed bone layers to the completely formed bone. The complete model consisted of approximately 30,000 nodes and 40,000 elements.

**Table 1:** Material properties of finite element model

		Young's Modulus (MPa)	Poisson's Ratio
**Vertebral Body**	**Cortical Bone** **Cancellous Bone**	**14 500** **400**	**0.3** **0.3**

**Growth Plate**	**Sensitive** **Newly Formed Bone** **Transition**	**12** **100** **300**	**0.4** **0.3** **0.3**

**Intervertebral Disc**	**Nucleus** **Annulus**	**2** **8**	**0.49** **0.45**

The spine models loading was set according to the data as reported by Schultz [27]*i.e.*, the spine loading increases from 14% to 57% of the body weight along the spine from T1 to L5, with the 2.6% body weight increase between succeeding vertebrae. Two distinct spinal loading techniques were programmed. Under the gravitational approach, the loading direction was maintained axially to simulate forces which, when coupled with the selected boundary conditions (T1 restricted in the transverse plane and L5 limited in all degrees of freedom) provided appropriate spinal stability. Another loading technique, the "follower load", was alternatively simulated in a fashion that the resultant forces from cumulative loads on each vertebra was maintained tangential to the curvature of the spine in the sagittal plane and remained axial in the coronal plane. This follower load type proved to allow improved spinal stability under ex-vivo spinal loading [28] and previously utilized in spinal finite element analyses [29]. Therefore, the vector direction gravitational loading approach remained constant throughout the iterative process described below whereas the direction follower load method regulated itself in order to maintain sagittal tangential loading under each numerical cycle. Both these approaches were explored separately. The model was built and computed using ANSYS 11.0 finite element package (ANSYS Inc., USA).

### Model of spinal growth

According to Stokes [16], the extent of actual growth *G *was calculated as the product of normal (uniform) growth *G*_*m *_and the regularizing term, which was represented by the scaled difference between the stress on the growth plate (σ) and that under the regular condition σ_*m *_(β = parameters representing the sensitivity of bone growth modulation to the applied stress):

In order to represent the global result of the growth mechanism in each individual vertebra, the local deformation of the elements of the newly formed bone layer within the growth plate was simulated as in previous finite element analyses of scoliotic spines [23]. An iteration of vertebral body growth consists of four consecutive steps, namely applying forces, measuring stresses in each element of the growth plates, calculating growth, and updating the geometry. The Cobb angles, kyphosis angle, and the lordosis angle were output after each iteration.

### Validation

The validity of the developed modeling platform to comply with scoliotic progression was explored using patient data. Three patients were selected with different curve types: Lenke type-1A, Lenke type-2A, and Lenke type-3C, with no significant alteration in sagittal spinal alignment *i.e.*, kyphotic curves between 20 and 35 with less than 5 degrees modification over time. These patients previously underwent an annual radiographic follow up of 3, 2, and 2 years respectively. The formerly described simulation methods were performed utilizing regular adolescent growth rates (*G*_*m*_) of 0.8 mm/year and 1.1 mm/year in thoracic and lumbar spines respectively [30]. Starting from initial patient curves, the model was constructed and its ability to corroborate with patient data was deemed successful if curve patterns were replicated within 5 degrees for the Cobb angles.

### Simulation of Different Vertebral Column Growth Patterns

**Figure 2:**
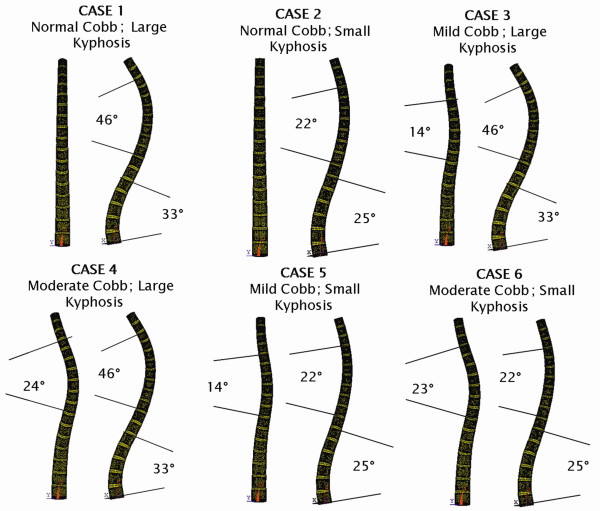
**Six initial states for the FEM model of spinal column (postero-anterior and sagittal views): Case 1 was reconstructed from a normal subject, and Cases 2~6 were generated from Case 1**.

Based on initial geometry of the patient-specific model, five other spine geometries with different kyphosis angles and lateral curves were generated by varying the spatial orientations of the vertebral bodies and intervertebral discs (Figure 2). Based on these six geometrical models, the corresponding finite element models were generated. The variety of spinal configurations allowed a detailed analysis of the influence of varying growth profiles on spinal alignment.

**Figure 3:**
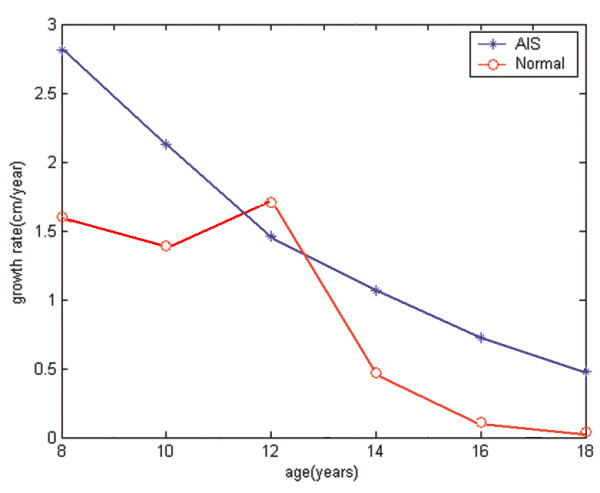
**The curve of growth velocities in AIS patients and normal controls during age 8 to 18**.

Vertebral growth between the ages of 8 to 18 was modeled iteratively with intervals of 2 years on all six initial models illustrated in Figure 2. Parameter values adopted in the simulation were *β *= 0.4 MPa^-1 ^and σ_*m *_≈ 0.5 MPa, as in relevant published studies [31,32]. The value of *G*_*m *_was set according to the growth velocity reported by Stokes [15]. By taking the baseline spine length at age of 8 as 36cm [33], the growth velocity was converted from the percentage value per year to the actual values in centimeters per year, as shown in Figure 3. In order to test the effect of different vertebral column growth patterns, in all six cases, the only difference in the simulation between AIS and normal spinal growth was the difference in growth profiles (*G*_*m*_).

### Sensitivity analyses

Several steps were undertaken to ensure that the model corroborated with reality while behaving in a robust fashion. The growth algorithm utilized in this analysis was acquired from in-vivo experimentation [34] while its application utilizing finite element analysis to explore progressive scoliotic spines has previously been demonstrated [21,23]. The longitudinal stresses measured in the intervertebral disc L5 showed agreement with in-vivo measurements [35]. Furthermore, in order to explore the influence of the adopted numerical assumptions on the results, several sensitivity analyses were performed. These additional simulations explored the influence of the selected growth constant (*β *= 0.4 to 0.6 MPa^-1^, a range of plausible physiologic values [36]), the loading configuration (gravitational and follower type spinal loading), and the magnitude of the growth velocities (*G*_*m *_= ± 15% of values reported in Figure 3).

## Results

**Figure 4:**
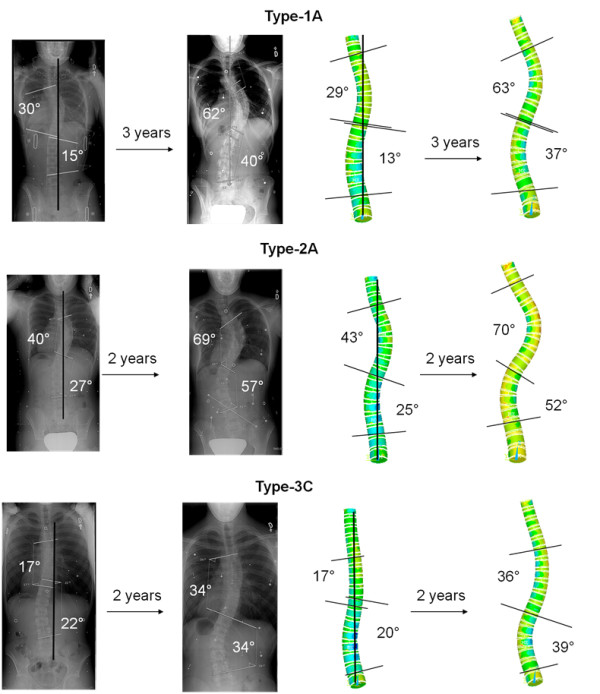
**Patient data and finite element model of growth simulation showing scoliotic progression agreement after 2 or 3 years**.

Preliminary validation analyses of simulated scoliotic progression proved positive in corroborating with patient specific progressive profiles. For each scoliotic type, the finite element model was able to agree with sequential patient data within 5 degrees for both lumbar and thoracic curves after 2 or 3 years of simulated spinal growth (Figure 4).

**Figure 5:**
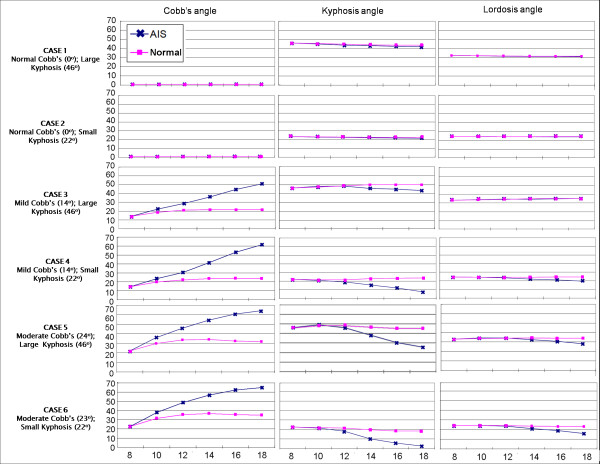
**Simulation results for all the six cases when AIS and normal growth profiles were adopted: the changes in Cobb, kyphosis and lordosis angles (vertical axes) as the age (horizontal axis) increases from 8 to 18**. The blue curves ("AIS") represent the angle changes when the AIS growth profile was adopted; the pink curves ("H") denote the results of the normal growth profile.

Figure 5 shows the simulation results in all the six cases for both AIS and normal growth profiles. The results from Cases 1 and 2 showed that when the initial coronal plane deformity was negligible: coronal Cobb angle, kyphosis, and lordosis angles remain fairly stable under both AIS and normal growth profiles. That is, when no lateral deformity is present no scoliotic curves presented themselves over ten years of simulated growth.

However, when an initial coronal deformity was present, both AIS and normal growth profiles resulted in increased lateral deformity. This progressive trend was significantly amplified when using the AIS growth profile. More specifically, after ten years of simulated growth cases 3, 4, 5, and 6 underwent Cobb angle increases with magnitudes respectively measured at 5.0, 4.3, 5.2, and 3.4 times larger using AIS growth profile compared to the normal one.

In addition to the augmented increase in the lateral deformity under the AIS growth profile, another finding suggest that the presence of an initial lateral deformity encourages a decrease in kyphosis under AIS growth profile while no such trends were observed with the normal growth profile. This observation held true despite the variety of explored initial kyphosis angles. Conversely, no significant changes were found in the lordosis angle of all the six cases.

The sensitivity analysis of *β *(0.4 to 0.6 MPa^-1^) magnified spinal Cobb angle amplitudes as *β *was increased. The final angle measures (after 10 years of simulated growth) were roughly doubled under a *β *of 0.6 compared to 0.4. However, relative differences between final measures of AIS and healthy growth rates remained as described above. That is, the relative increase in coronal Cobb (Cobb_AIS growth_/Cobb_normal growth _after 10 years of simulated growth), for cases 3 to 6, varied lightly between 1.85 to 2.61 and 1.79 to 2.86 when using a beta of 0.4 and 0.6 respectively. Therefore, it was consistently maintained that the AIS growth rate significantly encouraged additional scoliotic progression. Analysis of spinal loading (gravity force or sagittal plane follower load) also proved not to significantly alter the tendency of AIS growth to promote progression. To elaborate, the average Cobb angle increase initiated by the use of the AIS growth profile was between 1.85 to 2.61 and 1.50 to 1.88 when adopting gravity and sagittal follower loads respectively. Analysis of growth velocities (*G*_*m *_= ± 15%) altered magnitude of measured Cobb angles, to a lesser extent than factor *β*, and again, the relative comparisons were not significantly changed. More specifically, even when a 15% decrease in AIS growth rates was coupled with a 15% increase in normal growth velocities, final coronal Cobb angle related to AIS growth remained 1.8 times larger than that of the normal growth.

## Discussion

This study is the first to explore the influence of different vertebral growth patterns on AIS progression using state-of-the-art biomechanical modeling techniques. This research utilizes the "vicious cycle" notion of scoliotic progression under different spinal growth rates (accelerated AIS and normal). Simulation results of the finite element models, which were reaffirmed via sensitivity analyses, suggest that when an initial deformity is present, a faster AIS growth profile significantly encourages scoliotic progression in the coronal plane and decreases kyphosis in the sagittal plane. This result is consistent with the observations made between the height and angle velocities in AIS patients [37] and agrees with the tendency for scoliotic patients to adopt a reduced kyphosis [9,10]. Result from Case 2 also suggests that the presence of a small kyphosis angle cannot lead to scoliosis exclusive of an initial coronal deformity. Therefore, results suggest that the abnormal growth pattern of the anterior spine may play a secondary instead of a primary role in the development of AIS.

To investigate if the spinal deformity was incurred by the axial rotate on of each vertebral body, axial rotation angle of every vertebral body, with respect to a fixed global reference plane, was analyzed. Minor axial rotation (less than 5 degrees) was measured. This observation is consistent with the simulation results reported in the literature [21,38]. Therefore, it is perceivable that other mechanisms are involved in transverse plane deformations that include vertebral rotation, axial torsion, and rib hump.

A potential limitation of this analysis resides with the mere modeling of anterior spine growth. However, in a preceding study [20], it was shown that pedicle growth rate asymmetry (neuro-central growth plate) was neither able to independently generate a scoliosis nor to act in conjunction with other deformations to initiate scoliotic spinal curves. In addition, the cylindrical shape used to model the vertebral bodies may influence local stress distributions over the growth plates. However, this study seeks to draw comparative conclusions utilizing identical platforms while altering a single variable of interest (i.e. the growth rate) and, for the purpose of this analysis, such a factor was not deemed important. Moreover, assumptions that were considered to have important influence on the reported results were explored under complementing sensitivity analyses. Therefore, although the implemented numerical approach contains simplifications, sensitivity analyses suggest that adopted numerical techniques do not interfere with relative conclusion reported herein. Finally, as always in biomechanics, finite element modeling is a technique for simulating a mechanism of interest performed under logical assumptions rather than completely reconstructing reality. Therefore, results and conclusions of this study should be interpreted within the prescribed conditions. These modeling simplifications are not able to fully account for the functional limitations of the posterior elements and the coupling between loads in the different directions. However, resulting contact forces on facet joints might be more important in loading modes such as torsion, flexion/extension, and lateral bending as compared to compression, which may modify transmitted spinal loads. Thus, although not explored, these contact forces might potentially play a role in the scoliosis deformation process.

In the growth modulation equation, *G*_*m *_represents the growth rate (result of growth profile), *β *is the sensitivity factor and may be linked with the biological influences, and *σ *reflects mechanical factors (asymmetrical stress distribution). This is the first study that isolated and quantified the impact of growth profile (*G*_*m*_) on scoliotic progression, showing that the augmented G_m_, combined with an initial coronal deformity, will lead to progression of coronal deformity as well as decreasing the kyphosis angle in the spine.

The magnitude of the adopted parameters used in the analyses may have influenced the simulations results and therefore the conclusion. More specifically, although the sensitivity factor *β *was held constant between the explored cases, *β *may vary with the patient age [39]. Moreover, AIS patients' progression is mostly concentrated immediately prior to puberty, which may be related to circulatory hormones [40] (i.e., estrogen and melatonin). Such notions feed the speculation that, due to a variation in biological factors, the sensitivity factor *β *may be influenced by the disturbed growth plate mechanotransduction. For this reason, a sensitivity analysis exploring the influence of this variable was performed. The outcome of such study was encouraging and demonstrated that the identified association between scoliotic progression and increased growth velocity was robust. Although the current modeling settings were determined according to existing literature, one must recognize the possibility of inter-patient variability. Nevertheless, the developed finite element platform effectively allowed for the isolation of growth rate influence to implicitly explore its impact on the progressive profiles of scoliotic patients.

## Conclusions

This study presented the biomechanical comparison of accelerated AIS and normal vertebral body growth patterns on scoliotic progression using finite element modeling. Result of this analysis suggests that amplified AIS growth velocity could indeed lead to the supplementary progression of scoliosis and thus pose as a progressive risk factor. Whether the documentation of patient growth profiles has any clinical predictive value for curve progression deserves further investigations.

## Competing interests

The authors declare that they have no competing interests.

## Authors' contributions

LS, DW, and MD performed the finite element analyses. All authors were involved in drafting the manuscript or revising it critically. All authors have given final approval of the version to be published.

## Abbreviations

AIS: adolescent idiopathic scoliosis; FEM: finite element model
